# Radiobiological effects of the interruption time with Monte Carlo Simulation on multiple fields in photon beams

**DOI:** 10.1002/acm2.13110

**Published:** 2020-12-03

**Authors:** Hisashi Nakano, Daisuke Kawahara, Satoshi Tanabe, Satoru Utsunomiya, Takeshi Takizawa, Madoka Sakai, Hirotake Saito, Atsushi Ohta, Motoki Kaidu, Hiroyuki Ishikawa

**Affiliations:** ^1^ Department of Radiation Oncology Niigata University Medical and Dental Hospital Niigata Japan; ^2^ Department of Radiation Oncology Institute of Biomedical & Health Sciences Hiroshima University Hiroshima Japan; ^3^ Department of Radiological Technology Niigata University Graduate School of Health Sciences Niigata Japan; ^4^ Niigata Neurosurgical Hospital Niigata Japan; ^5^ Department of Radiology and Radiation Oncology Niigata University Graduate School of Medical and Dental Sciences Niigata Japan

**Keywords:** external photon beam, interruption time, microdosimetric kinetic model, Monte Carlo simulation, sublethal damage repair

## Abstract

**Purpose:**

The interruption time is the irradiation interruption that occurs at sites and operations such as the gantry, collimator, couch rotation, and patient setup within the field in radiotherapy. However, the radiobiological effect of prolonging the treatment time by the interruption time for tumor cells is little evaluated. We investigated the effect of the interruption time on the radiobiological effectiveness with photon beams based on a modified microdosimetric kinetic (mMK) model.

**Methods:**

The dose‐mean lineal energy y_D_ (keV/µm) of 6‐MV photon beams was calculated by the particle and heavy ion transport system (PHITS). We set the absorbed dose to 2 or 8 Gy, and the interruption time (τ) was set to 1, 3, 5, 10, 30, and 60 min. The biological parameters such as α_0,_ β_0,_ and DNA repair constant rate (a + c) values were acquired from a human non‐small‐cell lung cancer cell line (NCI‐H460) for the mMK model. We used two‐field and four‐field irradiation with a constant dose rate (3 Gy/min); the photon beams were paused for interruption time τ. We calculated the relative biological effectiveness (RBE) to evaluate the interruption time's effect compared with no interrupted as a reference.

**Results:**

The y_D_ of 6‐MV photon beams was 2.32 (keV/µm), and there was little effect by changing the water depth (standard deviation was 0.01). The RBE with four‐field irradiation for 8 Gy was decreased to 0.997, 0.975, 0.900, and 0.836 τ = 1, 10, 30, 60 min, respectively. In addition, the RBE was affected by the repair constant rate (a + c) value, the greater the decrease in RBE with the longer the interruption time when the (a + c) value was large.

**Conclusion:**

The ~10‐min interruption of 6‐MV photon beams did not significantly impact the radiobiological effectiveness, since the RBE decrease was <3%. Nevertheless, the RBE's effect on tumor cells was decreased about 30% by increasing the 60 min interruption time at 8 Gy with four‐field irradiation. It is thus necessary to make the interruption time as short as possible.

## 1 Introduction

When photon beams irradiate cells, the cells' DNA is damaged, affecting the cells' life and death. Some damaged cells can recover from the damage, via sublethal damage repair (SLDR).^1^, ^2^ SLDR begins within minutes after photon‐beam irradiation and completes within 4–6 hrs.^1^, ^2^ With SLDR, lessening photon beams' cell‐killing effect on relative biological effectiveness (RBE) may be possible by increasing the irradiation dose‐delivery time.^3^, ^4^ The dose‐delivery time's effects on radiobiological effectiveness were evaluated with single‐field photon beams and a microdosimetric kinetic (MK) model;^5^, ^6^, ^7^, ^8^, ^9^ the surviving fraction (SF) was increased by prolonging the dose‐delivery time, and the relative biological effectiveness was decreased.^10^

Fractionated irradiation with multiple‐field is used clinically to cover radiation targets with the prescribed dose but prevent toxicity to surrounding normal tissues.^11^ When multiplefield irradiation is applied, the prescribed dose is not administered consecutively; there is an interruption time (an interval between radiation fields) used so that irradiation interruption occurs at several sites/operations, for example, the gantry, collimator, couch rotation, and patient repositioning within each field.^12^, ^13^, ^14^ Unlike single‐field photon beam irradiation, treatment times are prolonged by interruption times.

The modified MK (mMK) model considers various irradiation methods with photon beams^15^, ^16^, ^17^ and better estimates the SF at higher radiation dose range compared to the MK model. With the mMK model, the SF of more clinically relevant conditions can be better estimated due to the fractionated irradiation. SLDR's effects during interruption times on the RBE have been studied using the mMK model, as a method similar to those used previously, especially for particle therapy.^18^, ^19^, ^20^, ^21^ Few studies have examined the effects of multiple‐field photon beam irradiation with interruption times on the RBE. Since photon therapy is more commonly used than particle therapy, such data may have a great impact. We evaluated the interruption time's effect on radiobiological effectiveness by setting several interruption times between multiple‐field photon beams, using the mMK model.

## 2 Methods

### 2.1 Monte Carlo simulations calculated by the PHITS

**Fig. 1:**
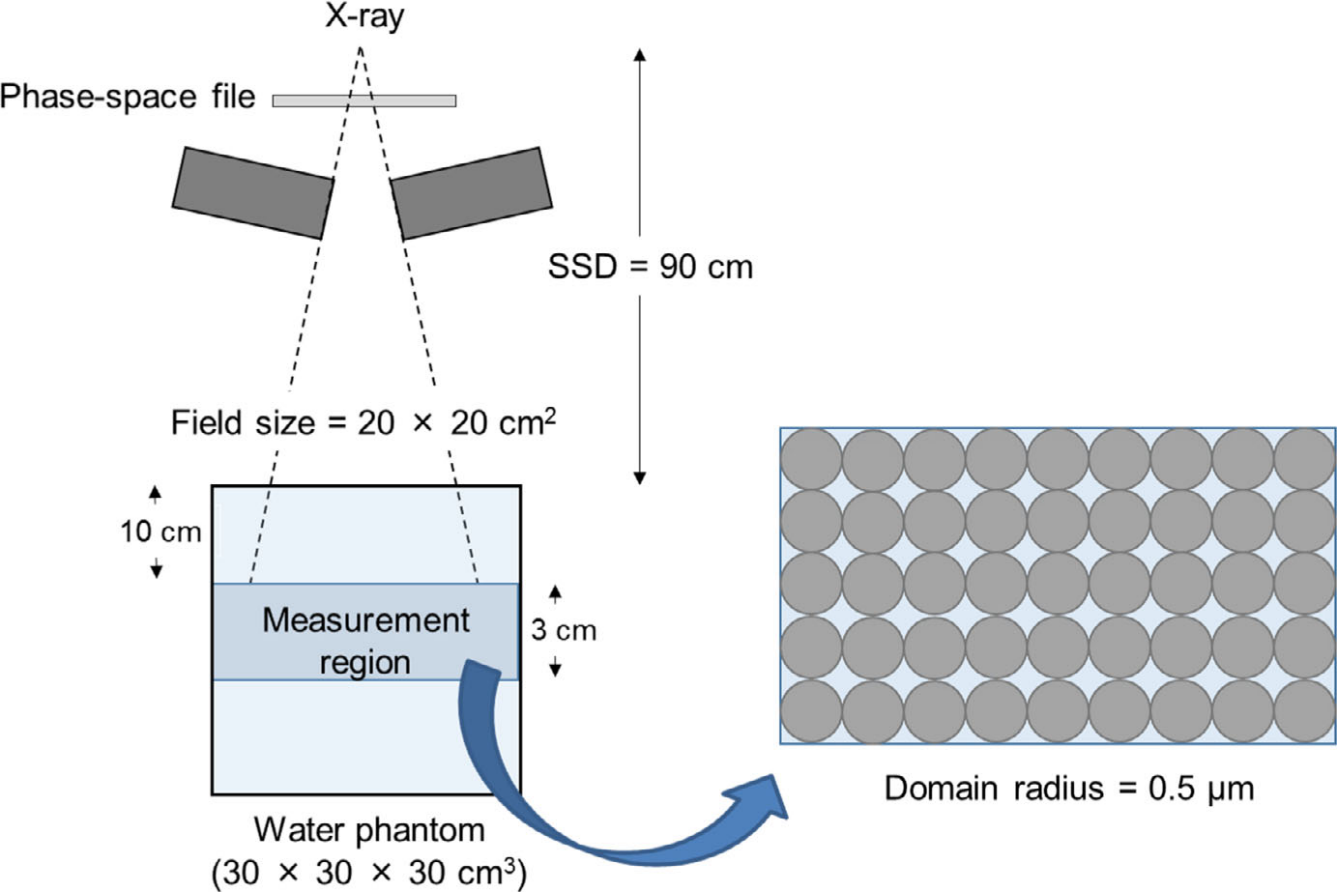
Irradiation geometry for the Monte Carlo calculations with 6‐MV photon beams. The domain radius was 0.5 μm in the 3‐cm‐wide measurement region in a water‐equivalent phantom.

Monte Carlo simulations code the particle and heavy ion transport code system (PHITS) and can deal with photons, electrons, positrons, neutrons, and heavy ions.^22^, ^23^, ^24^, ^25^ We used PHITS ver. 3.02 and the International Atomic Energy Agency phase‐space file of the Varian TrueBeam linear accelerator (Varian Medical Systems, Palo Alto, CA, USA) to calculate the dose‐mean lineal energy y_D_ of 6‐MV photon beams. The below phase‐space files were made using BEAMnrc, which is built on the EGSnrc platform.^26^ We transferred these phase‐space files created by BEAMnrc to the PHITS system to calculate the dose distribution. The irradiation geometry default settings were used for the PHITS calculations with 90‐cm SSD, 20 cm × 20 cm field size (Fig. 1), with a 10‐cm‐deep measurement point, 3‐cm calculation width in the water‐equivalent phantom, and 0.5‐μm domain radius. The dose‐mean lineal energy y_D_ ^23^, ^24^, ^25^ was calculated as:

(1) $$y=\frac{\varepsilon }{l}$$

(2) $$y_{D}=\frac{\int y^{2}f\left(y\right)dy}{\int yf\left(y\right)dy}=\frac{\int yd\left(y\right)dy}{\int d\left(y\right)dy}$$

where ε = the energy deposited in a domain, *l* = mean chord length, y = lineal energy, f(y) = the lineal energy' probability density, and d(y) = the lineal energy' dose distribution.

The dose‐mean lineal energy y_D_ of 6‐MV photon beams was calculated as a function of y‐yd(y): Eq. (2). We used the average y_D_ value obtained by simulating the calculations of the SF and biological effectiveness in the mMK model.

### 2.2 The SF and RBE calculations for interrupted 6‐MV photon beams using the mMK model

The mMK model considered various irradiation schemes with photon beams.^17^ The equation that determines the mMK model is:

(3) $$‐l_{n}S=\sum_{n=1}^{N}\left[\left(\alpha_{0}+\gamma \beta_{0}\right)D_{n}+\beta_{0}D_{n}^{2}\right]+2\sum_{n=1}^{N‐1}\sum_{m=n+1}^{N}\left\{\beta_{0}\left[e^{‐\left(m‐n\right)\left(a+c\right)\tau_{n}}\right]\right\}D_{n}D_{m}$$

(4) $$\gamma =\frac{y_{D}}{\rho \pi r_{d}^{2}}$$

(5) $$D=\dot{D}T$$

(6) $$\left(a+c\right)=\frac{\ln2}{T_{1/2}}$$

**Fig. 2:**
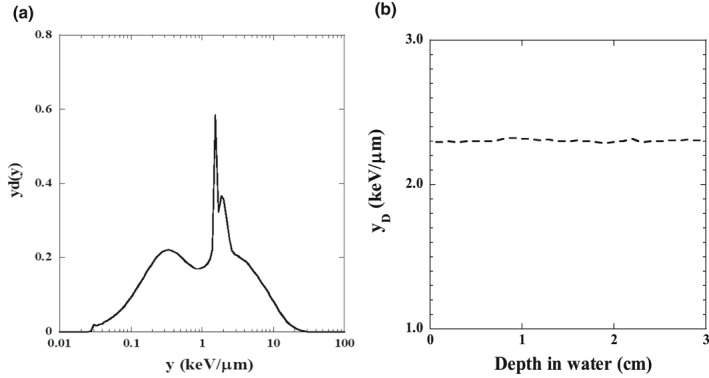
Four‐field photon beams using the mMK model, considering the photon beams' interruption time.

The D_n_ (D_m_) is defined the absorbed dose in n_th_ (m_th_) field at a regular interval [Gy], and α_0_, β_0_, and (a + c) are cell‐dependent constants. The parameter ρ is the domain's density, and r_d_ is the domain' radius (0.5 μm). The y_D_ is the dose‐mean lineal energy [keV/μm], $\overset{\cdot }{D}$ is the dose rate [Gy/min], and T is the dose‐delivery time [min]. The potentially lethal lesions (PLL) repair rate of the cell‐specific value (a + c) indicates the constant rate of DNA repair equated with the first‐order rate λ,^27^, ^28^ which we calculated using the DNA repair half‐time T_1/2_.^27^, ^28^ We defined the interruption time of each field as τ_n_ [min]. We used the biological parameters of the human non‐small‐cell lung cancer cell line NCI‐H460 to determine the mMK model parameters. King et al. reported the biological parameters α_0_ and β_0_ using an linear‐quadratic (LQ) model.^29^ Each of the DNA repairs occurred at a different rate constant, and the DNA repair time was calculated using the DNA repair half‐time T_1/2_.^30^, ^31^ Figure 2 illustrates four‐field photon beams using the mMK model, considering the photon beams' interruption time. Figure 2 also shows that the deformed Eq. (3) can be considered the interruption time (min) for both the linac pulse interval and each field interval. We calculated the SF and RBE using the mMK when the interrupted photon beams had two‐field and four‐field irradiation. The NCI‐H460 cells' absorbed dose varied at 2–8 Gy. The cells were irradiated with the absorbed dose D_1_ at the constant dose rate $\overset{\cdot }{D}$ (3 Gy/min). The irradiation was interrupted for a specified time (τ_1_). Second, the absorbed dose D_2_ was irradiated at the constant dose rate $\overset{\cdot }{D}$ (3 Gy/min). The cells' absorbed dose was D_1_ at this constant dose rate; the irradiation was interrupted for a specified time (τ_2_). Third, the absorbed dose D_3_ was irradiated at the same constant dose rate; the irradiation was interrupted for τ_3_. Finally, the absorbed dose D_4_ was irradiated at the same constant dose rate. The summed values of interruption times τ_1~n_ were 1, 3, 5, 10, 30, and 60 min. We divided the absorbed dose into two for the two‐field irradiation and into four for the four‐field irradiation

The RBE of the interrupted photon beams was defined using the instantaneous irradiation (τ = 0) with no interruption of the photon beams, as a reference (Eq. 7).^18^, ^32^, ^33^ Other cell‐specific values (a + c) (1.0 and 2.0) were used to assess the DNA repair date (a + c) values' effects on the RBE.

(7) $$RBE=\left[\frac{D_{\tau =0}}{D_{\tau }}\right]={\left(\frac{\sqrt{\alpha_{\tau =0}^{2}+4\beta_{\tau =0}S_{\tau =0}}‐\alpha_{\tau =0}}{2\beta_{\tau =0}}\right)}^{‐1}\cdot \left(\frac{\sqrt{\alpha^{2}+4\beta S}‐\alpha }{2\beta }\right)$$

## 3 Results

### 3.1 The interruption time's effects on the SF with two‐ and four‐field irradiation in the mMK model

**Fig. 3:**
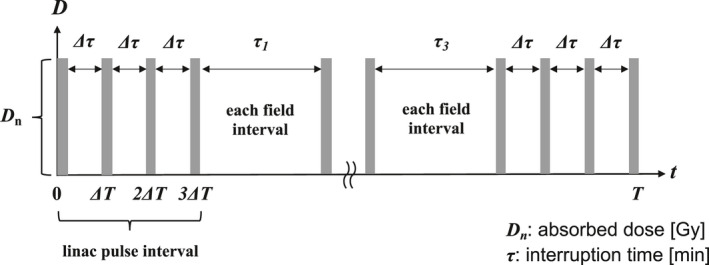
The dose‐mean lineal energy y_D_ as a function of y‐yd(y) for 6‐MV photon beams at 10‐cm‐deep in a water‐equivalent phantom (a). Relationship between the depth (3 cm from the measurement point in the phantom) and the dose‐mean lineal energy y_D_ (b).

**Fig. 4:**
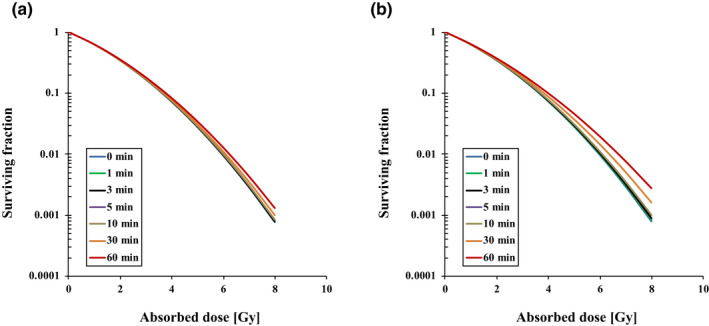
The interruption time's effect on the SF with two‐field (a) and four‐field (b) irradiation with various interruption times.

The relationships between the measured position's depth in the water‐equivalent phantom and the y_D_ are illustrated in Fig. 3. Changing the water depth hardly affected the y_D_ value; we thus averaged the y_D_ over a 10–13‐cm depth range. Table 1 lists the y_D_ average and standard deviation values for photon beams. Figure 4 illustrates the various interruption times' effects on the SF with two‐ and four‐field irradiation. The SF was higher with the interruption time's increase in both irradiation types. The difference between SFs with four‐field irradiation was emphasized with interruption times at 10, 30, or 60 min. The interruption time's effect was greater as the absorbed dose rose.

**Table 1:** Calculation parameters for the mMK model obtained using NCI‐H460 cells.

Parameter	Value
α_0_ (Gy^‐1^)	0.21 ± 0.16
β_0_ (Gy^‐1^)	0.07 ± 0.03
a + c (h^‐1^)	0.46
y_D_	2.34 ± 0.01
ρ (g/cm^3^)	1.00
$r_{d}\left(\mu m\right)$	0.5
D (Gy)	2, 8
$\overset{\cdot }{D}$ (MU/min)	300
Field number	2, 4
τ (min)	1, 3, 5, 10, 30, 60

### 3.2 The interruption time's effect on the RBE with two‐ and four‐field irradiation

**Fig. 5:**
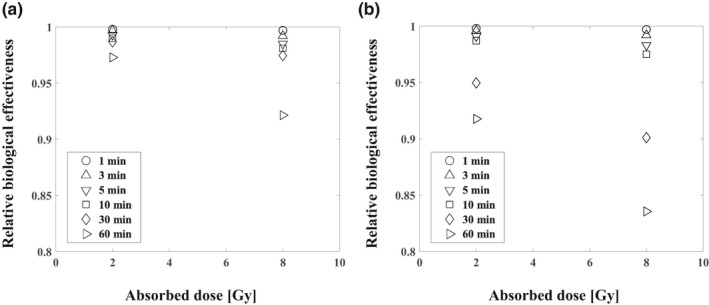
The interruption time's effect on RBE with two‐ (a) and four‐field (b) irradiation with various interruption times.

Figure 5 shows the RBE of 6 MV photon beams in the two‐ and four‐field irradiations and different interruption times. With two‐field irradiation, the RBE was decreased to 0.998, 0.997, 0.993, 0.990, 0.987, and 0.973 for interruption times τ = 1, 3, 5, 10, 30, and 60 min with 2 Gy, and 0.997, 0.992, 0.985, 0.981, 0.975, and 0.921 with 8 Gy, respectively. With four‐field irradiation, the RBE was decreased to 0.998, 0.996, 0.991, 0.987, 0.950, and 0.918 with 2 Gy, and 0.997, 0.992, 0.983, 0.975, 0.900, and 0.836 with 8 Gy, respectively.

### 3.3 Relationship between the DNA repair constant rate (a + c) and the RBE with various interruption times

**Fig. 6:**
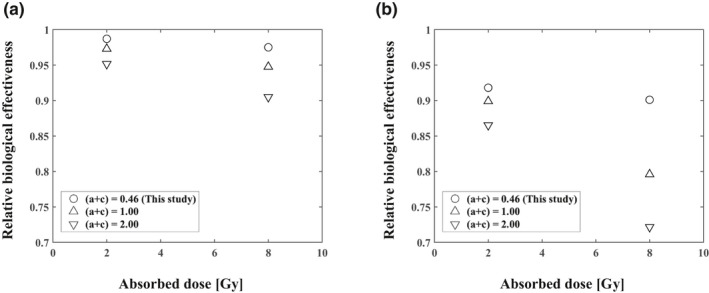
RBE as a function of the cell‐specific repair rate (a + c) with τ = 10 min (a) and τ = 60 min (b) (four‐field irradiation).

Figure 6 reveals that the RBE value depended on the DNA repair constant rate (a + c) with τ = 10 and 60 min in four‐field irradiation. Table 2 summarizes the relationship between the repair constant rate and the RBE at 2 and 8 Gy with various interruption times. Notably, the RBE was affected by the repair constant rate value: the larger the (a + c) and the longer the interruption time, the greater the RBE decrease.

**Table 2:** The effect of RBE as a function of the cell‐specific repair rate (a + c) for 2 and 8 Gy with various interruption time using two‐ and four‐field irradiation.

	Interruption time (min)					
	1	3	5	10	30	60
(a + c) = 1.00						
2 Gy						
Two‐field	0.999	0.997	0.995	0.990	0.973	0.951
Four‐field	0.997	0.991	0.986	0.973	0.934	0.899
8 Gy						
Two‐field	0.998	0.994	0.990	0.981	0.948	0.905
Four‐field	0.994	0.983	0.972	0.948	0.870	0.796
(a + c) = 2.00						
2 Gy						
Two‐field	0.998	0.994	0.990	0.981	0.951	0.920
Four‐field	0.994	0.983	0.973	0.951	0.899	0.865
8 Gy						
Two‐field	0.996	0.989	0.981	0.964	0.905	0.841
Four‐field	0.989	0.967	0.948	0.905	0.796	0.722

## 4 Discussion

We evaluated the RBE of 6‐MV photon beam irradiation with interruption times calculated from the SF using the dose‐mean lineal energy y_D_ and the mMK model. The dose‐mean lineal energy y_D_ was calculated by the PHITS, and the y_D_ was scarcely affected by changing the water depth. We thus conclude that the irradiation field's depth with various interruption times has no effect on the RBE.

The irradiation applied in radiotherapy can be interrupted due to a linear accelerator's mechanical problems, sometimes for a long term.^34^ Moreover, irradiation techniques such as a respiratory gating system used to attain tumor control for lung cancer require a large absorbed dose per fraction, a protracted dose‐delivery time, and a long interruption time.^10^, ^14^ Figure 5 provides the results of our calculation of the interruption times' effects on the RBE. A several‐minutes‐long interruption had no significant effect on the RBE within 3%, but a ≥10% reduction of the RBE occurred when the 8‐Gy four‐field irradiation was interrupted for 30 or 60 min. With a >30‐min longer interruption time resulted in a large RBE difference or cell SF difference caused mainly by SLDR. It may thus be necessary to shorten the photon beams' interruption as much as possible, since the RBE was decreased by prolonging the interruption time. Based on these results, we speculate that a prescribed dose taking the interruption time into account is required when a long irradiation interruption (>30 min) occurs.

We used NCI‐H460 cells to calculate the interruption time's effect. The RBE value was dependent on cell‐specific values (a + c) of the DNA repair constant rate τ = 10 and 60 min; Fig. 6), and the RBE was affected by the cell‐specific value (a + c); the larger that this value was, the greater the decrease in RBE was (Table 2). The RBE was maximum decrease about 30% under the condition of the 60 min interruption time, four‐field irradiation, and largest (a + c) values at 8 Gy in this study (Table 2). The cell‐specific value of the DNA repair indicates the recovery from tumor sublethal damage, depending on the tumor‐cell type.^35^ Further studies were necessary to evaluate how the interruption time affect search type of tumor cell.

Kawahara et al. evaluated the RBE with various interruption times and two‐field irradiation for human salivary gland tumor cells.^36^ The RBE of 8 Gy with a 10‐min interruption was decreased by ~4.0%;^36^ that is a large reduction compared to the lung cancer cells examined herein. It is thus necessary to more accurately evaluate the interruption time's effects for each tumor‐cell type, to simulate clinical conditions.

Several study limitations should be addressed. We simulated the interruption time's effects by using an mMK model and tumor‐cell parameters derived from *in vitro* experiments. It is necessary to verify the SF and RBE calculated and derived using the measured values. We evaluated the interruption time's radiobiological effect considering only the tumor SLDR, since the mMK model considered only SLDR. Other repair phenomena such as potentially lethal damage repair and repopulation were not considered. The relevance of tumor hypoxia and tumor reoxygenation occurring during the interruptions to the RBE was not evaluated.

## 5 Conclusions

The ~10‐min interruption of 6‐MV photon beams did not significantly impact the radiobiological effectiveness, since the RBE decrease was <3%. Nevertheless, the RBE's effect on tumor cells was decreased about 30% by increasing the 60 min interruption time at 8 Gy with four‐field irradiation. It is thus necessary to make the interruption time as short as possible. With a long interruption time, an escalation of the prescribed dose may be necessary.
